# Memoir on the Treatment of White Swellings by Compression

**Published:** 1841-10

**Authors:** 


Art. II.—*De la Compression contre les Tumeurs Blanches des parties dures.* Par le Docteur De Lavacherie, Professeur de Clinique Externe à l'Université de Liège, Membre Correspondant de la Société de Médecine de Gand, &c.—*Gand*, 1839. 8vo, pp. 99.

*Memoir on the Treatment of White Swellings by Compression.* By Professor Lavacherie, of Liège.—*Ghent*, 1839.

The author admits that pressure has been tried by many surgeons in "white swellings," but imagines that no surgeon hitherto has applied it so universally as himself, nor to such formidable cases, nor as the sole remedy. He applies compression to every stage and form of the disease; to acute cases as well as to chronic ones; where there is but slight enlargement, and where the tumefaction of the soft parts is very great; to simple enlargement, and to disorganization of the cartilages and ligaments; to inflammation of the bone, and to superficial or deep caries of the articular surfaces with or without fistulous communications with the joints themselves. By compression he has cured affections which had been pronounced incurable, and for which amputation had been proposed. When compression does not cure, he has found that it contributes powerfully to stop the progress of disease, and especially to relieve pain, which in many instances is intolerable. Even when applied where there is copious purulent secretion from deep-seated disease, strong pressure diminishes the secretion, and in all cases the pus makes room for itself between the skin and the plaster, however great has been the degree of compression employed.

M. Lavacherie makes pressure by means of strips of adhesive plaster, (emplâtre agglutinatif) firmly applied round the diseased joint. At first he used bandages with starch, but these did not exert sufficient pressure, and he found that the contact of adhesive plaster with the skin produced none of the irritation he at first feared. He applies the first straps in circles over that part of the joint which is most swollen; the other straps more obliquely, in order that they may be flat. Having covered the whole joint he then applies a roller over the whole limb. When the bones themselves are enlarged much stronger pressure may be used than when the soft parts are the principal seat of the disease. Stiffness is generally complained of, which ceases after the first twenty-four hours; after which time the bandage applied over the lower part of the limb to prevent stagnation of the circulation may be removed. In order to be useful, the pressure should not produce actual pain; the sufferings of the patient should gradually diminish, and in some hours cease. If the pain increases, the bandage has been badly applied, and should be removed and reapplied. In general the comfort produced is such that as soon as the apparatus ceases to exert pressure, the patient requests it may be renewed. This is generally the case after a few days, and it should then be reapplied. In many of the reported cases, the strapping was renewed every fortnight.

Thirteen cases are given, which M. Lavacherie has thus arranged:—6 perfect cures of white swellings, arrived at a stage reputed incurable; 3 equally bad cases so much improved that cures may be shortly expected; 3 other cases in which improvement is so marked that cures may be expected; one case much more severe than the others, where there is a sensible improvement. The conclusion drawn by the author from these facts is, that white swellings are amenable to treatment even at an advanced stage, and that compression is the best remedy; for if it does not cure, it delays the progress of the disease. He is convinced that formerly he has amputated limbs which he could now save. Even when from constitutional disease palliative treatment alone is advisable, compression is the best means, as it stops the progress of slow hectic fever, by excluding the air.

It is almost needless to say that the treatment of white swellings by compression is no novelty in this country. Mr. Scott, of Bromley, has the credit of introducing it; and although he does not depend on simple pressure, yet surgeons generally have regarded the methodical and scientific way in which he applies his bandages, so as to produce constant, equable, and long-continued pressure, as the essential part of his treatment. If this is a new thing on the continent, our neighbours must be much behind us in the treatment of diseased joints. In our own practice we have tried simple compression by adhesive plaster, and seen it used by others, in aggravated cases where there were fistulous communications with deep-seated disease of the bones, but certainly not with the success of our author. Nevertheless, it is a remedy that should be tried, being both simple and often efficacious. If the rule is attended to, that any increase of pain is a proof that the pressure has been ill applied or is producing mischief; and if the patient is carefully watched, and the bandages at once removed, no ill effects can follow, and many limbs may be saved which are now amputated; for doubtless the general disbelief in the power of bone to repair itself when ulcerated often leads surgeons astray when they feel a piece of rough bone beneath their probes, and thus many are maimed for life unnecessarily.

